# Integration of Metabolite Profiling and Transcriptome Analysis Reveals Genes Related to Volatile Terpenoid Metabolism in Finger Citron (*C. medica* var. *sarcodactylis*)

**DOI:** 10.3390/molecules24142564

**Published:** 2019-07-15

**Authors:** Yaying Xu, Changqing Zhu, Changjie Xu, Jun Sun, Donald Grierson, Bo Zhang, Kunsong Chen

**Affiliations:** 1 Zhejiang Provincial Key Laboratory of Horticultural Plant Integrative Biology/Laboratory of Fruit Quality Biology, Zhejiang University, Hangzhou 310058, China; 2 Zhejiang Agricultural Technology Extension Center, Hangzhou 310029, China; 3 Plant and Crop Sciences Division, School of Biosciences, University of Nottingham, Sutton Bonington Campus, Loughborough, Leicestershire LE12 5RD, UK

**Keywords:** citrus, limonene, γ-terpinene, WGCNA, terpene synthase gene, transcription factor, RNA-Seq

## Abstract

Finger citron (*Citrus medica* var. *sarcodactylis*) is a popular ornamental tree and an important source of essential oils rich in terpenoids, but the mechanisms behind volatile formation are poorly understood. We investigated gene expression changes combined with volatile profiling of ten samples from three developing organs: flower, leaf, and fruit. A total of 62 volatiles were identified with limonene and γ-terpinene being the most abundant ones. Six volatiles were identified using partial least squares discriminant analysis (PLS-DA) that could be used as markers for distinguishing finger citron from other citrus species. RNA-Seq revealed 1,611,966,118 high quality clean reads that were assembled into 32,579 unigenes. From these a total of 58 terpene synthase (TPS) gene family members were identified and the spatial and temporal distribution of their transcripts was measured in developing organs. Transcript levels of transcription factor genes *AP2/ERF* (251), *bHLH* (169), *bZIP* (76), *MYB* (155), *NAC* (184), and *WRKY* (66) during finger citron development were also analyzed. From extracted subnetworks of three modules constructed by weighted gene co-expression network analysis (WGCNA), thirteen *TPS* genes and fifteen transcription factors were suggested to be related to volatile terpenoid formation. These results provide a framework for future investigations into the identification and regulatory network of terpenoids in finger citron.

## 1. Introduction

Plants produce a range of small molecule volatile and non-volatile organic compounds called secondary metabolites that are not essential to the growth and development of the producing organism. But enable them to adapt to their living environment. Terpenoids constitute the largest group of volatile secondary metabolites [1]. They are made from the basic C5 building blocks, isopentenyl diphosphate (IPP) and dimethylallyl diphosphate (DMAPP) and are synthesized by the mevalonate (MVA) pathway in the cytosol and the methyl-erythritol phosphate (MEP) pathway in the plastid [2]. IPP and DMAPP are condensed by head to tail reactions to form geranyl diphosphate (GPP), farnesyl diphosphate (FPP) and geranylgeranyl diphosphate (GGPP) [3]. Terpene synthases (TPS) function to catalyze the terminal steps in these pathways and utilize substrates GPP, FPP and GGPP to form monoterpene (C10), sesquiterpene (C15), and diterpene (C20), respectively. The diversity of terpene synthase (TPS) gene members and their catalytic mechanisms generate extremely complex products [4]. Monoterpenes and sesquiterpenes are volatile at room temperature. They are important secondary metabolites with biological functions such as promoting stress resistance or acting as plant pheromones [5,6]. Moreover, monoterpenoids and sesquiterpenoids contribute to the characteristic aroma of fruits, including citrus fruits [7].

TPS families have been identified in many plants, for example Arabidopsis, tomato, grapevine, and apple [8,9,10,11]. Chen et al. comprehensively introduced the structure, function, and evolution of plant TPS [12]. The modern plant TPS family went through several evolutionary events such as gene duplication, functional differentiation, and divergence, and finally developed into seven subfamilies [8,13]. Dornelas et al. found 49 TPS genes from multiple citrus varieties by searching the CitEST database [14]. With the publication of the *Citrus sinensis* genome, Alquézar et al. described the first functionally characterized TPS family in citrus [15]. They identified seven genes which encode sesquiterpene synthases. Other terpene synthases from Citrus spp. have been functionally characterized in mandarin [16,17,18,19,20], lemon [21], sweet orange [22], and yuzu (*Citrus junos*) [23]. Recently, we have identified a sesquiterpene synthase associated with formation of bicyclogermacrene in finger citron [24].

Terpene biosynthesis changes during plant development. Progress has been made in identifying transcription factors (TFs) related to regulation of terpenoid synthesis. In citrus, CitAP2.10 [25] and CitERF71 [26] were reported to regulate the synthesis of the sesquiterpene valencene and monoterpenoid (*E*)-geraniol, respectively. MsMYB negatively regulates monoterpene synthesis in spearmint trichomes [27]. Nieuwenhuizen et al. found that mutation in the NAC binding region of TPS gene promoters in two kiwifruit species affect their monoterpene contents [28]. In cotton, GaWRKY1 up-regulates the expression of *CAD1*, leading to the accumulation of sesquiterpene phytoalexins, including gossypol [29] while overexpression of *OsbZIP79* causes a decrease in the accumulation of phytoalexin in rice cells [30]. Hong et al. found that AtMYC2 combines multiple plant hormone signals in the transcriptional regulation of sesquiterpene biosynthesis and activates the production of (*E*)-β-caryophyllene [31]. AaERF1 and AaERF2 positively regulate the expression level of *ADS* and *CYP71AV1*, leading to the accumulation of artemisinin [32,33].

Citron is one of the three primitive species of Citrus [34]. The carpels of finger citron split, causing a finger-like fruit shape [35]. It is a popular ornamental tree in oriental countries with dietary and pharmaceutical potentials [36,37]. Finger citron essential oils have antioxidant, antibacterial, antibiofilm activities [38,39,40] and they have been widely used in the food and perfume industry [41]. The chemicals isolated from finger citron essential oils are terpenes, terpene derivatives, and various volatile substances such as higher alcohols, aldehydes, ketones, and esters [38]. The most abundant components of finger citron essential oils are limonene and γ-terpinene [39,40]. Compared to other Citrus species, *C. medica* (citron) has a unique volatile profile [25,42,43] and according to González-Mas et al., it produces higher amounts of γ-terpinene, β-pinene or camphene, while lacking some compounds, especially non-terpenoid aldehydes [7].

Information on finger citron genes related to production of volatiles is still very limited, however, but now the availability of the *C. medica* genome provides essential reference for the investigation into gene function [34]. In the present study, we utilized metabolomic and transcriptomic methods to explore terpenoid biosynthesis and regulation in finger citron. Volatile profiles from developing finger citron organs were evaluated using GC-MS. Characteristic volatile compounds were identified by multivariate strategies. Also, the expression patterns of TPS, and TFs AP2/ERF, bHLH, MYB, NAC, and WRKY were described. We used weighted correlation network analysis (WGCNA) to uncover the correlations between terpene content and expression levels of specific genes [44]. This method has been successfully applied in mining potential TFs in grapevine and sugarcane [45,46] and elucidating the coordinated regulation of compounds in tea plants [47]. This strategy reconstructed subnetworks with high connectivities, presenting gene targets for functional characterization. This information will contribute to development of the citrus industry and provide information for future breeding programs.

## 2. Results

### 2.1. Volatile Profiling of Finger Citron

**Figure 1:**
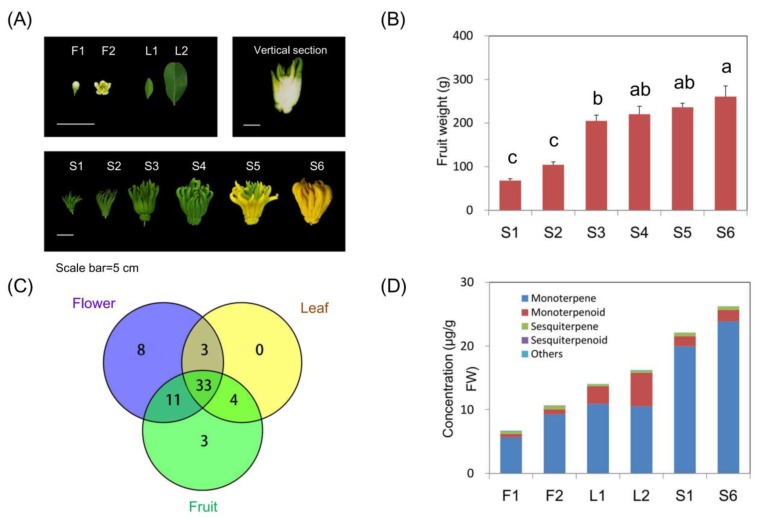
Major volatile classes from finger citron. (**A**) Photos of flower, leaf and fruit. (**B**) Changes in fruit weight during ripening. (**C**) Venn diagram of volatiles detected in three organs. (**D**) Concentration of major volatile classes in developing organs. F1, flower bud; F2, full flower, L1, young leaf; L2, mature leaf; S1–6, fruit developing stages 1–6. Scale bar = 5 cm. Significant differences between groups were detected by Duncan’s test at significant level of 0.05.

In order to obtain comprehensive information about volatile components of finger citron, flowers, leaves and fruits at different developmental stages were subjected to SPME-GC-MS analysis (Figure 1A). Finger citron fruit weight increased from 68.33 g to 260.77 g during development (Figure 1B). A total of 62 volatiles were detected in finger citron throughout these developmental stages (Figure 1C and Table S1). Major volatile classes of finger citron included 17 monoterpene hydrocarbons, 23 oxygenated monoterpenes, 13 sesquiterpene hydrocarbons, one oxygenated sesquiterpene, and 8 others including aldehydes, alkanes, ketones, and olefins (Table S1). Among these 62 volatiles identified, 33 chemicals (53.2%) were detected in all three organs (flower, leaf, and fruit). A total of eight chemicals (12.9%) were only detected in flower. Three chemicals (4.8%) were specifically produced by fruit and no unique volatiles were detected in finger citron leaf (Figure 1C). Monoterpene was the most abundant class, accounting for 64.82% to 90.97% of the total content, followed by monoterpene derivative accounting for 6.84% to 32.47% (Figure 1D). Finger citron accumulated volatile compounds during organ development, with the highest content of volatiles detected in fruit, followed by leaf and flower. Among all the volatiles detected, the most abundant chemical was limonene (3.88 mg/g to 16.73 mg/g), followed by γ-terpinene (0.92 mg/g to 7.22 mg/g) (Table S1).

**Figure 2:**
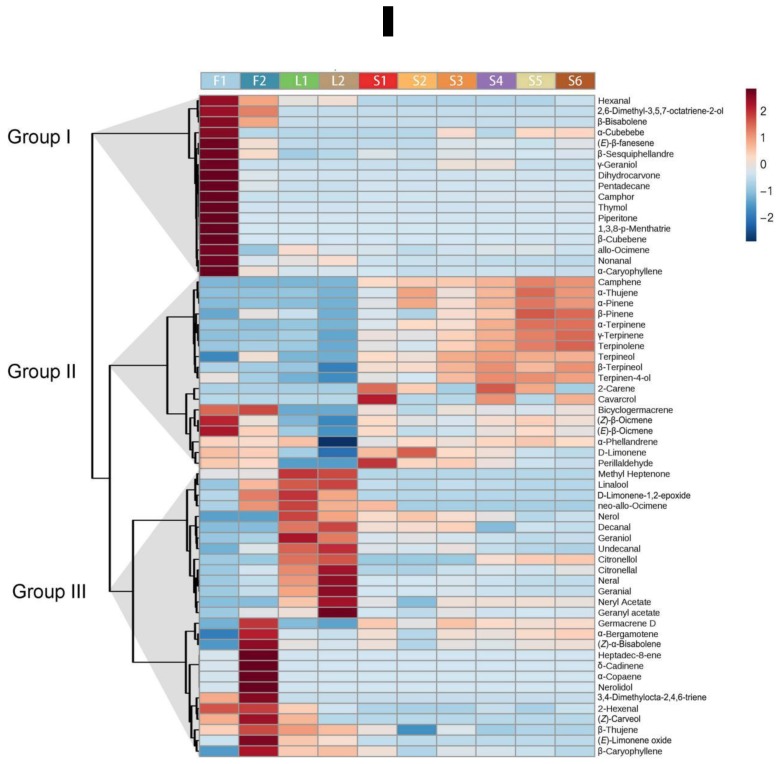
Hierarchical clustering and heat map visualization of changes in volatiles identified in developing finger citron. The left side of the heat map represents hierarchical clustering based on Pearson correlation. The color scale (−2 to 2) is shown on the right; red represents high content, blue represents low content. F1, flower bud; F2, full flower, L1, young leaf; L2, mature leaf; S1–6, fruit developing stages 1–6. Hierarchical clustering and heat map were constructed using MetaboAnalyst 4.0.

A heat map was constructed to provide an overall view of volatiles detected in finger citron organs at different developmental stages (Figure 2). In brief, volatiles could be clustered into three groups based on their profiles in finger citron organs. Group I volatiles were enriched in young leaf (F1). Volatiles in group II were enriched in fruit, including the two most abundant compounds, limonene and γ-terpinene. A total of 20 volatiles from group II were mainly detected in fruit and the contents of 17 volatiles accumulated during fruit development. In contrast, the content of the major volatile limonene tended to decrease during finger citron fruit ripening, declining from 16.73 mg/g to 13.41 mg/g (Figure 2 and Table S1). Group III consisted of 13 volatiles that mainly accumulated in fully opened flowers. The most notable change was observed for β-caryophyllene, whose contents increased more than 90-fold during flower maturation (Table S1).

### 2.2. Identification of Key Volatiles

**Figure 3:**
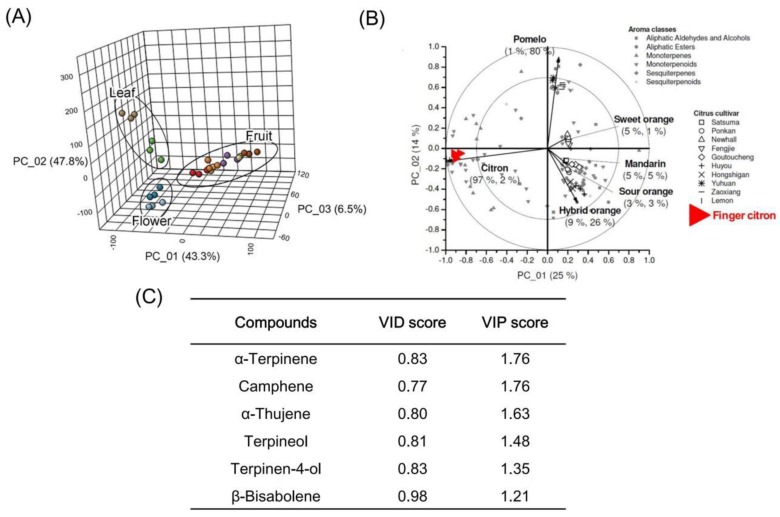
Volatiles ranked by variable identification (VID) and variable importance in projection (VIP) scores. (**A**) partial least squares discriminant analysis (PLS-DA) 3D score plot of finger citron using volatiles as variables. (**B**) PLS bi-plots of eleven *Citrus* varieties from six species. (**C**) Key volatiles ranked by VID and VIP scores. VID scores were calculated using Unscrambler vs 10.1 according to Shen et al.; VIP score was obtained by PLS-DA using online tool MetaboAnalyst 4.0.

Variation in contents of volatiles observed in Figure 2 prompted us to investigate if finger citron organs could be distinguished based on volatile profiles. A PLS-DA model was constructed to explore the separation of organs using volatiles as variables. Permutation and cross validation tests validated the significance of the treatment differences with *p* < 0.01 (Figure S1A,B), indicating that the obtained PLS-DA model is reliable. A separation of the finger citron leaf, flower, and fruit was observed in the PLS-DA score plot (Figure 3A) with no points overlapped. In order to identify biomarkers for discriminating finger citron samples, variable importance in projection (VIP) scores were calculated for each volatile. It has been reported that variables with value exceeding 1.0 play important roles in the PLS-DA discriminant process [48]. A total of 24 volatiles with VIP > 1.0 were identified (Figure S1C), indicating these were volatiles responsible for clustering and separation of finger citron samples. Among the top 10 compounds, nine were monoterpenoids, including γ-terpinene, α-terpinene, camphene, terpinolene, α-pinene, α-thujene, β-pinene, terpineol, and β-terpineol. Compared to flower and leaf, the concentrations of these monoterpenoids were higher in fruit, and they tended to accumulate during fruit ripening (Figure 2, Table S1). For volatiles mentioned above, terpinene isomers γ-terpinene and α-terpinene were the top two volatiles with high VIP values of 1.78 and 1.76, respectively.

Our previous study observed distinct volatile profiles of finger citron fruit compared to various other Citrus species and varieties [25]. PLS analysis was used in order to distinguish the six species by their volatile profiles (Figure 3B). The first two PLS-factors were able to explain 99% variance of citron relative to pummelo, hybrids, sour orange, mandarin, and sweet orange and the clustering results were in agreement with classic Citrus taxonomy. Volatile compounds that have important roles in discriminating citrus fruit samples were identified by Shen et al. and the VID-coefficients were calculated [25]. As an arbitrary threshold, a VID coefficient with absolute value of 0.70 was taken [49]. By comparing the VID-coefficient and VIP values for each volatile compound, 6 volatiles with VID score exceeding 0.70 and VIP score exceeding 1.0 were observed (Figure 3C). These volatiles are suggested to be characteristic compounds of finger citron separating it from other Citrus species (Figure 3).

### 2.3. A Transcriptome Atlas of Finger Citron

In an attempt to understand the changes in gene expression causing metabolite diversity during development, we sequenced the RNA libraries extracted from flower, leaf and fruit. Information about RNA-Seq quality is summarized in Table S2. The correlation coefficients of tested samples were listed in Figure S2. The correlation of the three biological replicates is above 0.7, with false discovery rate (FDR) value < 0.01 indicating the reliability of sampling. The relatively low correlation between organs indicates a selectively expression pattern of finger citron genes. A total of 1.5G clean data was generated, 95.80% of which was successfully mapped to the reference genome of C. medica. We assembled 32,579 genes, which were expressed in finger citron. For functional annotation, unigenes were searched against the Gene Ontology (GO) knowledgebase and the Kyoto encyclopedia of genes and genomes (KEGG). About 76% of the unigenes were assigned to GO terms and 42% of them were annotated with KEGG entries. This information establishes the foundation for further investigation into target genes. Fragments per kilobase of exon model per million mapped reads (FPKM) values were calculated to represent gene expression levels and the gene number and FPKM distribution are summarized in Figure S3. Most genes were expressed in the 0.3~3.57 and 3.57~15 intervals (Figure S3). In general, flowers had the most genes expressed and fruit had the least. As mentioned above, plant terpenoids are synthesized from two independent pathways and the upstream genes and their functions are well studied [2]. Hence, we screened key structural genes involved in terpene biosynthesis by KEGG annotation. The values of their expression fold changes are listed in Table 1.

Table 1 shows the DEGs in MEP and MVA pathways during organ development. In total, 12 DEGs were identified. No gene transcripts changed significantly during flower development. In the MEP pathway, two DXSs (catalyzing the formation of 1-deoxy-D-xylulose 5-phosphate), Cm152440, and Cm165980 were significantly down-regulated in fruit. DXS/Cm152440 was down-regulated in leaf while another DXS/Cm104830 showed an opposite trend. MDS/ Cm051360 and HDS/ Cm131750 were up-regulated in fruit and flower, respectively. In the MVA pathway, there were four genes differentially expressed: two were down-regulated in leaf and two up-regulated in fruit. During fruit development, the expression level of HMGR, which is considered as the rate limiting enzyme in the MVA pathway [50], was up-regulated by 5.5-fold.

### 2.4. Changes in Transcript Levels of TPS Gene Family

Terpene synthases catalyze the final pathway steps converting the universal substrates GPP, FPP, and GGPP into terpenoids. There are many members of the TPS family and their complex catalytic mechanisms contribute to the terpenoid diversity in the plant kingdom [4]. A total of 58 *TPS* genes were identified from finger citron based on KEGG annotation. A schematic diagram of TPSs gene structure (Figure S4) shows that finger citron *TPS* gene lengths ranged from about 200 bp to 16,000 bp. Some of these sequences are predicted to be inactive partial fragments in comparison to characterized TPSs in tomato and sweet orange [9,15].

**Figure 4:**
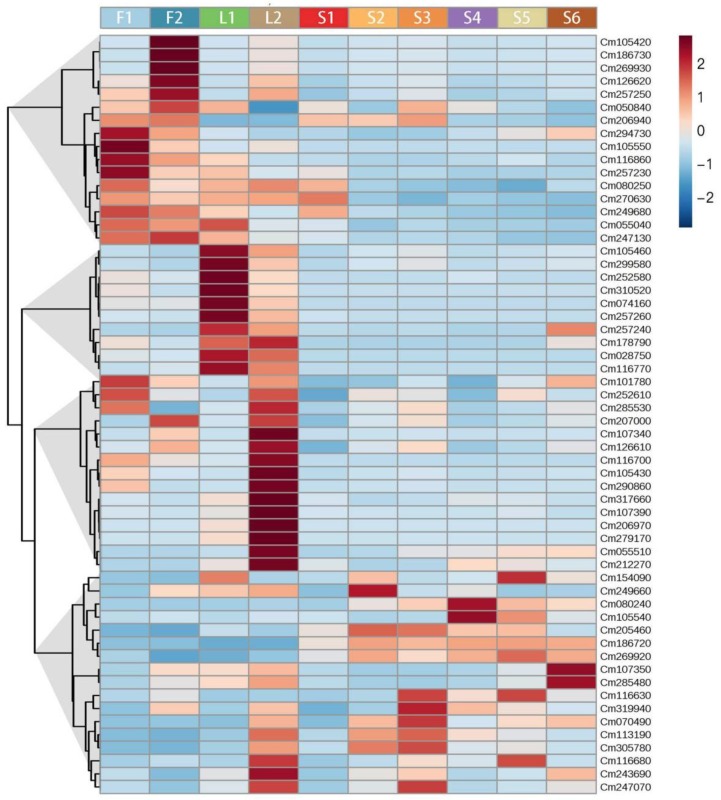
Hierarchical clustering and heat map visualization of changes in *TPS* gene expression level in developing finger citron organs. F1, flower bud; F2, full flower, L1, young leaf; L2, mature leaf; S1–6, fruit developing stages 1–6. The left side of the heat map represents hierarchical clustering based on Pearson correlation. The color scale (−2 to 2) is shown on the right; red represents high content, blue represents low content. Hierarchical clustering and heat map were constructed using MetaboAnalyst 4.0.

In order to provide an overview of expression profiles of the *TPS* gene family, their transcript levels were presented and visualized as a heat map (Figure 4). As found for volatiles in Figure 2, the *TPS* genes in Figure 4 were also clustered into groups based on their expression patterns, named TPS-Cluster1 to 4. For flower, genes in TPS-Cluster1 were mainly accumulated (Figure 4). The expression of six *TPS* genes (Cm105420, Cm186730, Cm269930, Cm126620, Cm257250, and Cm050840) increased during flower development, while four genes (Cm294730, Cm105550, Cm116860, and Cm257230) decreased. Genes from TPS-Cluster2 and TPS-Cluster3 were predominantly expressed in young and mature leaves, respectively. These two clusters together contain 25 genes, accounting for 38% of *TPS* gene family members. For TPS-Cluster4, genes were mainly expressed in fruit. During fruit development, transcript levels of two *TPS* genes (Cm186720 and Cm269920) increased markedly from S1 to S2 and then remained constant afterwards. High transcript levels of *TPS* genes Cm107350 and Cm285480 were detected in ripe fruit at S6 stage (Figure 4). The spatial and temporal expressing pattern implies the diverse function of TPSs and the regulating mechanism behind terpenoid metabolism, although extremely complex, can now be probed thanks to the developing of modern techniques.

To evaluate expression pattern based on RNA-Seq, RT-qPCR analysis was performed. Four fingers citron TPS genes were randomly selected for gene expression analysis during fruit ripening. As shown in Figure S5, RT-qPCR results matched the expression pattern produced by RNA-Seq. These results indicated that the RNA-Seq data of the present study were accurate and reliable. 

### 2.5. Changes in Transcript Levels of Transcription Factors

The transcript levels of *TPS* genes were regulated by a number of different TFs. So far, six TF families in plants associated with terpenoid metabolism have been identified, including AP2/ERF [25,26,32,33], bHLH [31], MYB [27], NAC [28], WRKY [29], and bZIP [30]. Their gene IDs are summarized in Table S3. Changes in the transcript levels of these six TF families from developing finger citron tissues were analyzed and presented as heat maps (Figure S6). The AP2/ERF family with 251 members was the largest, followed by the NAC family with 184 members and bHLH family with 169 members. As shown in Figure S5, TFs from different families had unique expression patterns.

For the AP2/ERF family, members were grouped into clusters according to expression profiles in developing tissues of finger citron (Figure S6). There were 88 (35%) *AP2/ERFs* mainly expressed in developing fruit, where nine members had the highest expression level in ripe fruit at the S6 stage. For flowers, 36 *AP2/ERFs* had the most abundant transcripts in full flower stage at F2. For finger citron leaves, higher transcript levels of 18 *AP2/ERFs* were observed in young leave L1 than mature leave L2 (Figure S6).

Among the 169 members in the bHLH family, 58 genes were mainly expressed in fruit, 43 genes in flowers and 68 genes in leaves (Figure S6). For fruit, 11 *bHLHs* had the highest expression level in young fruit at the S1 stage. During flower development, expression of 21 *bHLHs* increased from F1 to F2, while 22 members decreased. Of the *bHLHs* mainly expressed in leaves, 58 genes had higher transcript abundance in young leaves L1 than mature leaves L2 (Figure S6).

A total of 76 bZIP members were identified in finger citron, where 31 (41%) members were mainly expressed in leaves (Figure S6). For MYB family, 58 (37%) of the total 155 family members were enriched in flower tissue. A total of 44 *MYBs* exhibited higher transcript levels in young flower at F1 (Figure S6). We observed that *NAC* genes were enriched in flowers, where 52 (28%) members were expressed mainly in young flowers (Figure S6). Nearly half of the *WRKYs* (27 out of 66 genes) were enriched in fruit tissues. All of these TF expression profiles, together with those of the *TPS* genes, were used for the gene co-expression analysis.

### 2.6. Analysis of Gene Correlation Network

**Figure 5:**
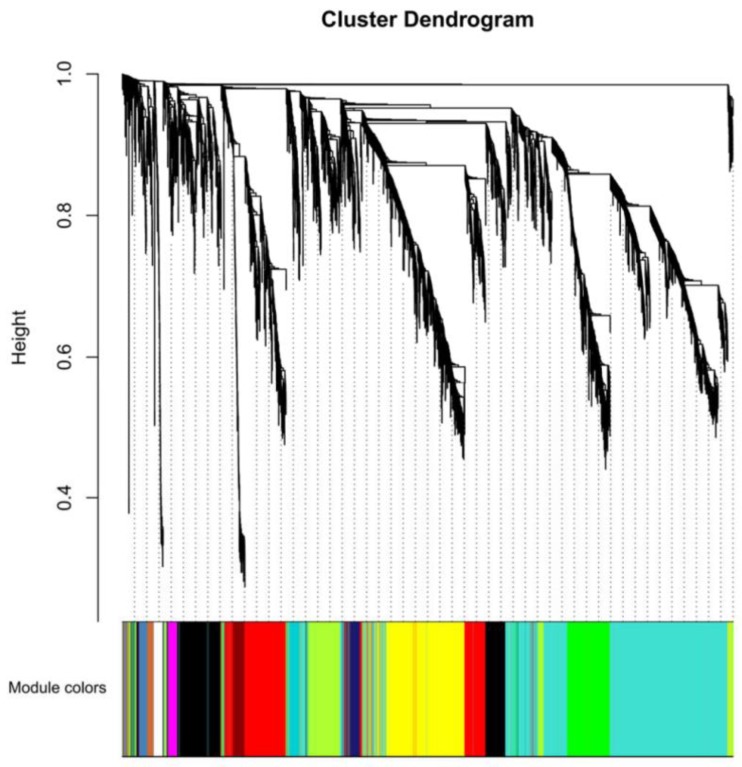
Clustering dendrogram of expressed genes. Gene modules were identified by dynamic hierarchical tree cut and shown in different colors. Height cut = 0.25, minimal module size = 30.

Both volatiles and genes showed dynamic changes in developing organs of finger citron. Weighted gene co-expression network analysis (WGCNA) was performed to get a better understanding of the relationships between them and their place in the complex signaling networks regulating terpenoid metabolism. A total of 45 gene modules from the dynamic hierarchical tree cut were obtained after a series of matrix transformation procedures and network construction (Figure 5). The largest module was Module Turquoise, containing 1954 genes, and the smallest, 30 genes each, was observed for Module Ivory and Module Floralwhite.

**Figure 6:**
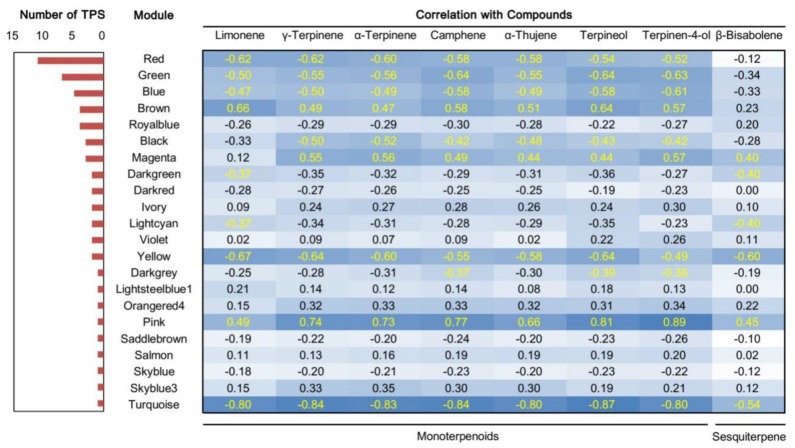
Correlation coefficients between terpene synthase (TPS), module and volatile compound. TPS numbers assigned to each module are list on the left. Correlation coefficients between volatiles and Module eigengenes are presented on the right with a color scale (−1 to 1). Darker blue color represents higher correlation. Coefficients with *p* < 0.05 are colored in yellow.

To evaluate the biological importance of the modules, the Pearson correlation between Module eigengenes and external sample traits (in this case, volatile concentrations) [44] were calculated and visualized in Figure 6. The most abundant two components (limonene and γ-terpinene) with the six characteristic components identified in Figure 3C were selected as interesting traits. The terpene synthase gene numbers in each module are shown on the left. In Figure 6, the 58 *TPSs* were assigned to 22 modules according to their transcript profiles. Finger citron *TPSs* occurred in more than twenty modules. This result is predictable because Figure 4 already implied a diversity of *TPS* expression patterns. The modules with the highest number of *TPS* genes were Module Red (11) Module Green (7) and Module Blue (5). In addition to the only sesquiterpene, β-bisabolene, the remaining volatiles showed similar correlation coefficients with a certain module. For example, the correlation coefficients between Module Red and seven monoterpenoids fell in a narrow interval (−0.62 to −0.52) and these monoterpenoids have similar accumulation patterns in finger citron tissues (Figure 2).

**Figure 7:**
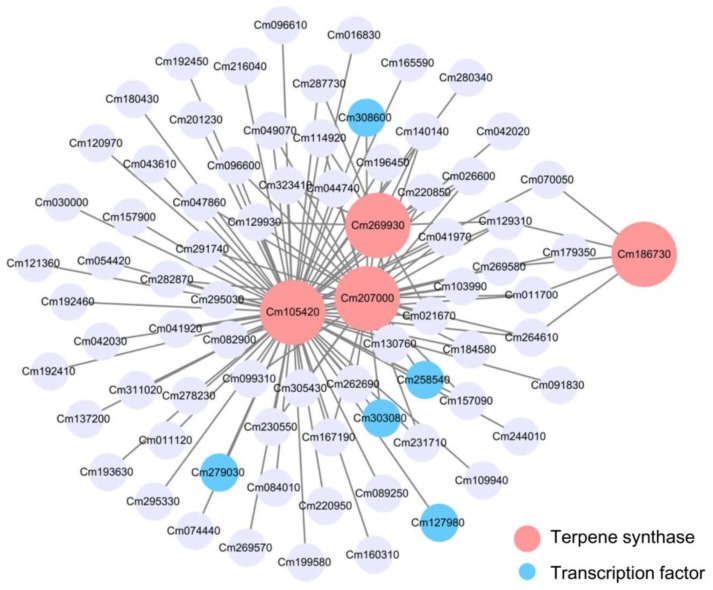
Gene co-expression subnetwork of Module Red. Network was reconstructed by edge weight cutoff = 0.35 and visualize by cytoscape. Gene IDs and annotations in Module Red subnetwork were listed in Table S4.

**Figure 8:**
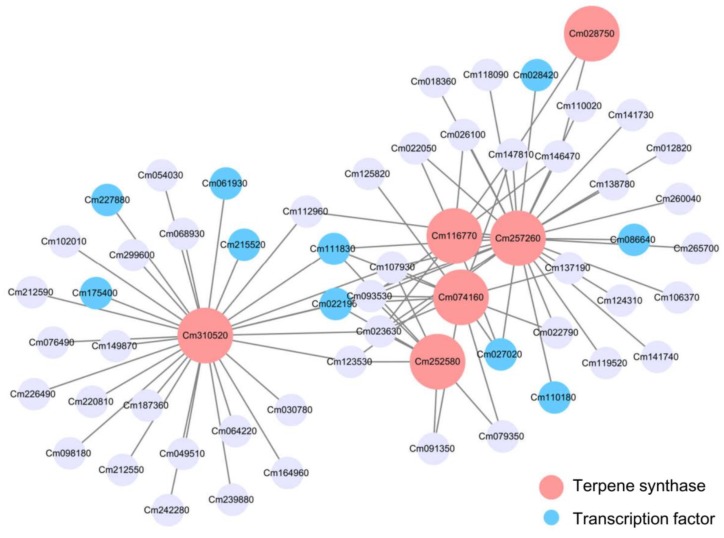
Gene co-expression subnetwork of Module Green. Network was reconstructed by edge weight cutoff = 0.35 and visualize by cytoscape. Gene IDs and annotations in Module Green subnetwork were listed in Table S5.

Module Red, Module Green and Module Blue were large modules, comprising 661, 707, and 1406 nodes, respectively. Connections of genes were hard to distinguish in the co-expression networks constructed on all nodes (Figure S7). So, to get a better view of genes strongly correlated to *TPS* genes, subnetworks were extracted from these three modules using TPSs as seed nodes. Subnetworks constructed from Module Red and Module Blue were visualized as Figure 7 and Figure 8, respectively. Although Module Blue is the largest of the three analyzed modules, no TF was identified in its subnetwork (Figure S8). In these subnetworks, two categories of genes which are the main focus of this work: *TPS* genes (red nodes) and transcription factors (blue nodes). Edges in figures indicate the interactions between the node gene and surrounding TFs in a module.

The subnetwork of Module Red contains four *TPSs*: Cm105420, Cm186730, Cm207000, and Cm269930 (Figure 7). Cm186730 was located on the right of the network, while the other three genes gathered in the center. A total of five TFs were observed in this subnetwork. As a member of NAC family, Cm303080 was connected to two *TPSs*, Cm207000 and Cm105420 (Figure 7). These two *TPS* genes were also connected to MYB family member Cm258540. The *TPS* gene Cm105420, transcript levels were also suggested to be related to transcription factor C2H2 family member Cm279030 and G2-like family member Cm127980. For *TPS* gene Cm269930, transcription factor CPP family Cm308600 was connected (Figure 7, Table S4). No transcription factors were observed to be connected to TPS Cm186730. Based the information in Table S4, Cm070050, Cm269580, and Cm264610 were all predicted to be polygalacturonase genes. This result indicates that, terpenoid metabolism and cell wall metabolism appear to be related in this specific module. 

There were six *TPSs* (Cm310520, Cm252580, Cm074160, Cm257260, Cm116770, and Cm028750) in the Module Green subnetwork. Four of them clustered together, including Cm252580, Cm074160, Cm257260, and Cm116770. *TPS* gene Cm257260 was connected to four TFs, including MYB family Cm028420, bHLH family Cm110180 and Cm027020, and ARR-B family Cm022190 (Table S5). For the other two *TPS* genes, Cm028750 was localized at the right edge, while Cm310520 on the left connected to 27 genes. Cm3102520, whose expression was suggested to be related to six TFs (Figure 8). These six TFs included MIKC_MADS family Cm111830, ARR-B family Cm022190, AP2 family Cm215520, ARF family Cm061930, bHLH family Cm227880, and HD-ZIP family Cm175400 (Table S5). Functions of other genes were also investigated (Figure S5). There were genes predicted to participate in secondary metabolism: Cm112960 and Cm107930. Cm093530 and Cm023630 was related to hormone signal transduction.

### 2.7. Phylogenetic Tree Analysis of Finger Citron TPS

**Figure 9:**
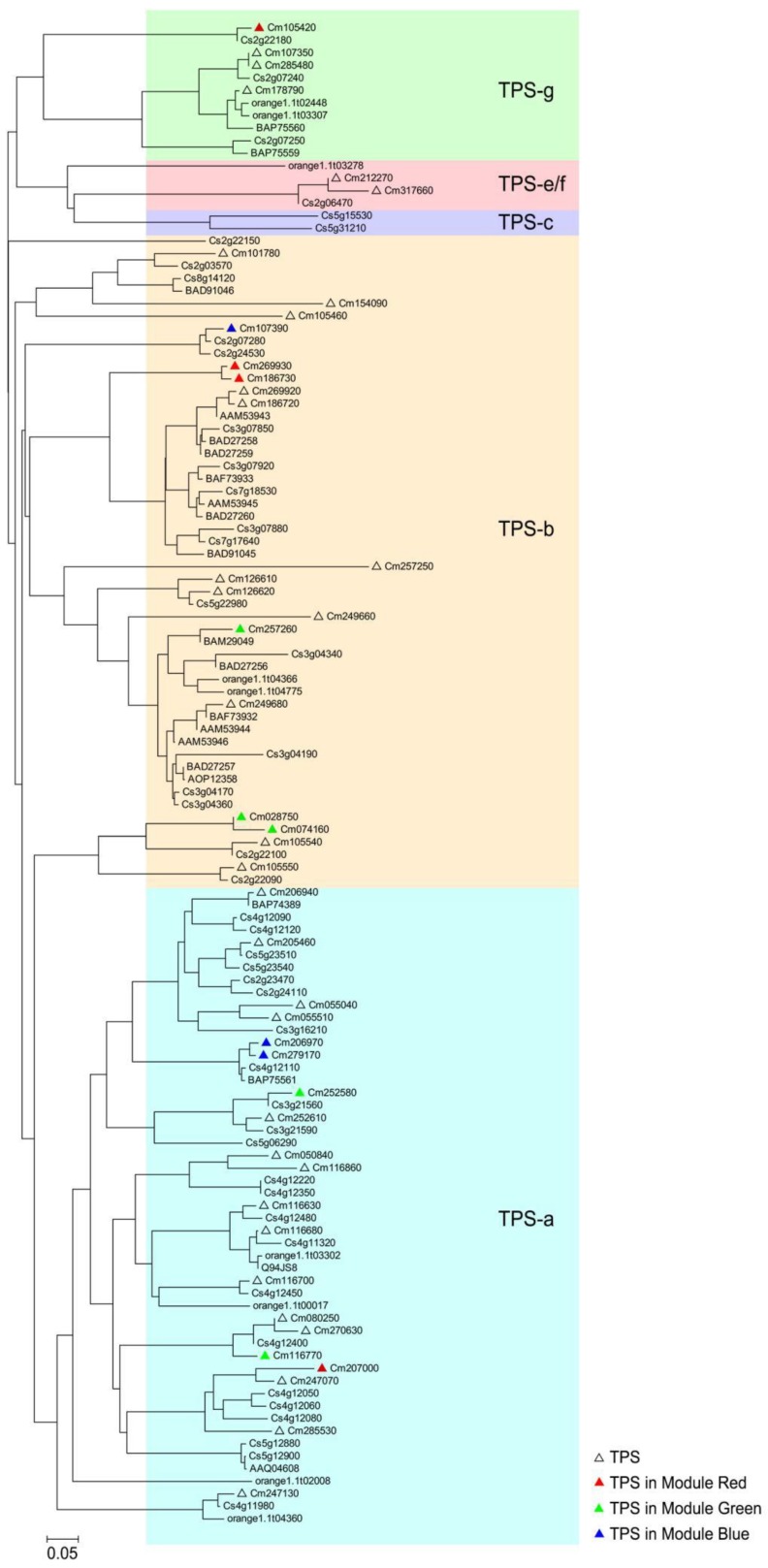
Phylogeny tree analysis of *Citrus* TPSs. Triangles represent finger citron TPSs, and filled colors indicate their location in modules shown in Figure 5. TPSs accession number of sweet orange and other *Citrus* species were reported by Alquézar et al. [15]. Sequence alignment was performed by ClustalX. Phylogeny tree was visualized by FigTree.

The functions of the TPSs remained unknown, so we constructed a phylogenetic tree based on their amino acid sequences (Figure 9) together with the Citrus TPS reported by Alquézar et al. In Figure 9, *TPS* genes in the phylogenetic tree were highlighted by red dots. Finger citron *TPS* genes were assigned to four subfamilies: 20 in TPS-a, 18 in TPS-b, 2 in TPS-e/f, and 4 in TPS-g. Genes in the TPS-a subfamily are predicted to catalyze the formation of sesquiterpenes [12]. There were 20 finger citron *TPS* belong to this subfamily. Genes in TPS-b usually encode proteins that function as monoterpene synthases. TPS-g is a clade closely related to TPS-b and there is evidence that monoterpenes, sesquiterpenes and diterpenes can be formed by genes from the TPS-g clade [10,51]. Despite their diverse functions, they share the common feature that all their products are acyclic. There are two finger citron TPSs that belong to the e/f clade. To get a more precise prediction of finger citron TPS function, sequence identities compared with characterized *TPS* genes from other *Citrus* species were calculated and are summarized in Figure S9. Since the correlated volatiles to Module Red and Module Green were monoterpenoids (Figure 6), attention was paid to genes in TPS-b and g subfamilies.

The extracted Subnetwork of Module Red contains 4 *TPS* genes. Cm269930 and Cm186730 shared 97.66% identity and they both belonged to TPS-b subfamily (Figure 9). Cm269930 was the hub gene, highly connected node, in Module Red. Sequence analysis showed that Cm269930 shared high amino acid sequence identity with *C. unshiu* γ-terpinene synthase BAD27258 (72.78%) and *C. limon* γ-terpinene synthase AAM53943 (71.01%) (Figure S9). Low sequence identity (29.26%–40.00%) was observed between Cm105420 and *Citrus TPSs* (Figure S9). Module Green contains 6 *TPSs*. TPS family member Cm310520 was ruled out for the phylogenetic analysis because of its inadequate length. Three members (Cm028750, Cm074160, and Cm257260) belong to TPS-b family. Sequence analysis showed that Cm257260 had 90.7% identity with *C. limon* (+)-limonene synthase AAM53944 and *C. jambhiri* limonene synthase BAF73932 (Figure S9). Cm116700 and Cm252580 belong to TPS-a family (Figure 9) and the Cm116700 sequence was 95.02% identical with *C. sinensis* sesquiterpene synthases Cs4g12350 (Figure S9). Cm028750 and Cm074160 sequence identities were less than 50% homolgous to *Citrus TPSs* sequences. For three *TPS* genes from Module Blue, Cm206970 and Cm279170 belong to TPS-a subfamily, while Cm107390 belong to TPS-g subfamily (Figure 9). Sequence analysis revealed that Cm206970 and Cm279170 had 96.17% and 97.76% identity with *C. unshiu* linalool synthase BAP75561, respectively (Figure S9). For Cm107390, sequence identities to characterized *Citrus TPSs* were less than 45%. These results indicated possible roles of finger citron TPSs from three Modules (Red, Green, and Blue) in biosynthesis of volatile terpenoids, although their functions need to be further investigated.

## 3. Discussion

Terpenoids are important components of citrus fruit volatiles. Finger citron has unique aroma, but the molecular mechanism of terpenoid biosynthesis remains unclear. In order to identify the characteristic compounds in this cultivar, we profiled the volatiles in 10 tissues from different finger citron organs. In total, 62 volatile chemicals were characterized, most of which were terpenoids. Consistent with previous studies in finger citron [38,39], the most abundant volatile is limonene followed by γ-terpinene. Variations in volatile compositions were also observed in different organs of *Citrus limon* and other *Citrus* species [52,53,54]. In the clustering heat map (Figure 2), we observed that most components are differentially expressed in different organs. The main component limonene, decreased when fruit matured and a similar pattern in finger citron was also observed by Wu et al. [40]. A decrease in limonene during fruit ripening was also detected in *Satsuma mandarin* [55,56]. We identified six characteristic components in finger citron (Figure 3). Apart from β-bisabolene, the other five volatiles are cyclic monoterpenoids. They are enriched in fruit and accumulated during fruit ripening. These components are suitable biomarkers that could be used to distinguish finger citron organs and finger citron extracts from those of other *Citrus* species.

We identified 58 members of the finger citron TPS family. This family size is smaller than in grapevine (152) and sweet orange (95), but similar to rice (57) and *Sorghum bicolor* (48) [12]. The largest clade of *TPS* genes found in finger citron, with 20 members, is the TPS-a clade. In sweet orange, TPS-a is also the largest clade [15]. In other plants where the TPS family has been analyzed at genome wide level, TPS-a clade is also the largest clade, including sweet orange [15], tomato [8], apple [11], and grape [10]. It has been suggested that more recent *TPS* gene duplications are indicated by an additional TPS-a clade [11]. Sesquiterpenes are major products produced by TPS-a enzymes, and the high number of TPS-a clade was suggested to be related to their possible role in plant developmental responses, such as defense [15]. RNA-Seq has the ability to simultaneously detect whole gene expression levels, and the availability of such a complete transcriptome profile has been a powerful tool to obtain insights into the molecular mechanisms underlying different conditions. In the present study, transcript levels of finger citron *TPSs* were widely detected in different finger citron organs during development. Similar observations were also made for apple and tomato TPS family in developing organs [9,11], suggesting important biological roles of TPS family members in plants.

To understand the regulation of finger citron terpenoid biosynthesis, expressed genes detected by RNA-Seq were connected by weighted gene co-expression network analysis (WGCNA). This provided a network made of nodes (genes) and edges (connections). Connections in the network were obtained based on gene co-expression data. This strategy has been applied in mining potential targets genes and transcription factors in plants [45,46,47]. In the present study, the top three modules enriched for *TPS* genes were selected for further analysis. Briefly, thirteen *TPS* genes were screened based on WGCNA, including four from Module Red (Cm269930, Cm207000, Cm105420, and Cm186730), six from Module Green (Cm028750, Cm116770, Cm257260, Cm074160, Cm252580, and Cm310520) and three from Module Blue (Cm206970, Cm107390, and Cm279170). Moreover, a total of 15 TFs were identified, including five from Module Red (Cm127980, Cm258540, Cm279030, Cm303080, and Cm308600) and 10 from Module Green (Cm022190, Cm027020, Cm028420, Cm061930, Cm086640, Cm110180, Cm111830, Cm175400, Cm215520, and Cm227880). These TFs consisted of 12 families, including ARR-B, bHLH, MYB, ARF, MYB, MIKC-MADS, HD-ZIP, AP2/ERF, G2-like, C2H2, NAC, and CPP. Compared to previous studies [25,26,28,57,58,59], our study revealed several novel TF families, ARR-B, ARF, MIKC-MADS, HD-ZIP, G2-like, and CPP, were associated with *TPSs* expression and volatile terpenoid formation. In the future, more experimental evidence is required to confirm the role of these candidate transcription factors.

Regarding to *TPS* genes identified based on WGCNA, six belong to TPS-a family, five belong to TPS-b and one belongs to TPS-g (Cm107390). TPS-a members were associated with sesquiterpene formation, TPS-b contribute to monoterpene biosynthesis, TPS-g is associated with monoterpene and sesquiterpene formation. Therefore, these identified TPS may have important roles in production of volatile terpenoids in finger citron. The *TPS* gene Cm310520 sequence is too short to include in the phylogenetic tree construction. Among these thirteen *TPS* genes, four (Cm105420, Cm028750, Cm074160, Cm107390) members had low sequence identity (<50%) to *Citrus TPS* genes with known functions. Relatively, high sequence identities (up to 97.76%) with characterized *Citrus TPS* genes were observed for eight TPSs of finger citron. As the final enzymatic step of MVA and MEP pathways, TPSs are responsible for the direct synthesis of terpenoids. However, in most case expression level of these *TPS* genes do not have a linear correlation with their product contents. The uncertainty arises for two main reasons. Firstly, a considerable number of TPSs are multi-product enzymes which produce several volatiles from a single substrate. Secondly, the replication and evolution of the TPS family has produced functioning isozymes express differently in time and space [4,20,60]. We have previously characterized a sesquiterpene synthase, *CmTPS1*, which has substrate selectivity, catalyzing the formation of 15 sesquiterpenes from FPP, with the main product bicyclogermacrene, followed by aromadenrene and elixene [24]. On the other hand, genes from separate clades have also evolved towards a similar function [8,12]. Therefore, more experiments are necessary to test functions of these 13-candidate finger citron TPS, such as overexpression in *E.coli* for enzymatic analysis in vitro and stable transformation for function analysis in vivo.

## 4. Materials and Methods

### 4.1. Plant Materials

Finger citron (*C. media* L. var. *sarcodactylis*) trees were grown in a commercial orchard in Jinhua, Zhejiang, China. Fruit at different developmental stages (S1-S6) were sampled at fixed time intervals: 55 days after bloom (DAB), 85 DAB, 115 DAB, 151 DAB, 182 DAB, and 212 DAB (Figure 1). Changes in fruit weight are shown in Figure 1B. Throughout the experimental period, average fruit weight increased steadily from 68.32 g to 260.77 g. Flower and leaf samples were collected at two developmental stages from the same orchard, with three biological replicates with three fruits each and 15 flowers and 20 leaves each [24]. Fruit of eleven *Citrus* cultivars from six species were collected at the commercial ripened stages as described by Shen et al. [25]. Materials were cut into pieces immediately after collecting, frozen in liquid nitrogen and stored at −80 °C.

### 4.2. Volatile Collection

Volatile compounds of three finger citron tissues were extracted using head space solid phase microextraction (SPME), using detailed procedures according to Xu et al. [24]. Frozen samples were ground into a fine powder under liquid nitrogen before use. In a 4 mL vial, 0.1 g tissue powder, 1 mL saturated NaCl solution, 10 μL 1-hexanol (0.1%, *v*/*v*) as internal standard were added. The vial was sealed with a PTFE-silicon septum and vortexed vigorously to mix well. The samples were incubated at 42 °C for 30 min, then extracted with an SPME fiber (50/30 DVB/CAR/PDMS) (Supelco Co., Bellefonte, PA, USA) for 30 min.

### 4.3. GC-MS Analysis

An Agilent 7890N gas chromatograph (GC) equipped with an HP-5 column (30 m × 0.25 × 0.25 μm, J & W Scientific, Folsom, CA, USA) coupled to an Agilent 5975C Network Mass Selective Detector (MS, inert XL MSD with triple-axis detector) were used for detection of volatiles. After extraction, volatile compounds were detached from the SPME fiber for 5 min at 250 °C by inserting the fiber into the GC injector. The GC oven temperature was started at 40 °C, increased to 70 °C at 3 °C /min, then to 130 °C at 1 °C/min, and finally ramped up to 230 °C at 15 °C/min. Carrier gas was helium with a flow rate at 1.0 mL/min. For detection of bicyclogermacrene, electron impact MS was used in selected ion current mode with an ionization energy of 70 ev. Mass spectra for compound comparisons were acquired over the range 35–350 *m*/*z* mass units. Volatile compounds were identified and checked by several methods. Chemicals were identified by comparing the electron ionization mass spectra of individual compounds with the standard spectrum of compounds from the NIST8.0 Mass Spectral Library (http://chemdata.nist.gov/). Kovats retention index of each volatile was calculated and compared to the same volatile identified by other researchers. Finally, when authentic standards were available, the retention time of each volatile was checked under exactly the same condition. Kovats retention index was calculated by injecting a C7–C21 linear alkane mixture standard, based on their molecular structure and retention time. Adding an internal standard as added as a reference helped in semi-quantitative determination of compounds.

### 4.4. Gene Expression Analysis by RNA Sequencing and Real-Time qPCR

For sequencing, total RNA was isolated from the ten finger citron tissues according to the protocol described by Xu et al. [24]. RNA was digested by Turbo-DNase (Ambion, Foster City, CA, USA). RNA quality was evaluated on a NanoPhotometer^®^ spectrophotometer (IMPLEN, München, Germany). The integrity and quantity of total RNA were assessed using a BioAnalyzer (Agilent Technologies, La Jolla, CA, USA). RNA-Seq was performed by LC-bio (Hangzhou, China) on the Illumina HiSeq 4000 platform. Raw reads obtained from RNA-Seq were pre-processed, adapters were trimmed; low-quality and shorter reads were removed. The *Citrus medica* genome was selected as reference for finger citron. Clean reads were aligned to the *C. medica* genome available online (http://citrus.hzau.edu.cn/orange/) by Hisat. Q20, Q30 and GC contents of the clean data were calculated. The expression profile of assembled gene was attached in Table S6. Functional annotation of genes was obtained from two public databases: KEGG (The Kyoto Encyclopedia of Genes and Genomes) and GO (Gene Ontology). Gene expression level was evaluated by fragments per kilobase of exon model per million mapped reads (FPKM). Three different RNA isolations were used as replicates for libraries construction and RNA sequencing.

To confirm the expression profiles obtained from the transcriptome data, the expression levels of four TPS genes were randomly selected for real-time qPCR (RT-qPCR) analysis. Total RNA was extracted as mentioned above. The first strand cDNA was synthesized using iScript™ cDNA synthesis kit (Bio-Rad, Hercules, CA, USA). The reaction system of qPCR was constructed using reaction mix SsoFast™ EvaGreen^®^ Supermix (Bio-Rad, Hercules, CA, USA) in a volume of 20 μL, according to the manufacturer’s instructions. The qPCR reaction program on CFX96TM Real-time system (Bio-Rad, Hercules, CA, USA) was set as follows: 95 °C for 3 min, 45 cycles of 95 °C for 10 s and 60 °C for 30 s, with a final melting curve step from 65 °C to 95 °C. Melting curve analysis for each pair of primers was performed to evaluate the PCR product specificity. Moreover, the PCR products were cloned into pMD18-T vectors (TaKaRa, Dalian, China) and sequence analysis confirmed the correct amplicons produced from each pair of primers. The primers were designed at the 3′UTR and sequences were listed in Table S7. At least three different RNA isolations and cDNA syntheses were used as replicates for the RT-qPCR. The relative expression levels were calculated as 2^△^^CT^ against internal reference citrus actin [24]. 

### 4.5. Sequence Analysis and Phylogenetic Tree Construction

Candidate *TPS* genes were screened by KEGG annotations and their sequence was confirmed by BLAST. *TPS* gene structure was visualized by DOMAIN/MOTIF Pattern Drawers from TBtools (v 0.58, South China Agricultural University, Guangzhou, China). A phylogenic tree of CmTPS was generated by ClustalX v 1.83 (Conway Institute UCD Dublin, Ireland) and visualized by MEGA7 v 7.0.14 [61]. *Citrus medica* Transcription factors were obtained by BLAST, taking *Citrus sinensis* transcription factors as reference. Reference sequences were downloaded from the public database PlanTFDB (http://planttfdb.cbi.pku.edu.cn/). TFs were divided into families based on their motifs. Sequence identity (%) of finger citron TPS with other *Citrus* species was calculated on MAFFT (https://www.ebi.ac.uk/Tools/msa/mafft/).

### 4.6. Gene Co-Expression Network Construction

Weighted gene co-expression network analysis was performed using R package [44]. A pairwise Pearson correlation matrix was created and further transformed into a weighted matrix. A topological overlap matrix was constructed with a threshold power of eight. A dynamic tree cut procedure (merge Cut Height = 0.25, min module size = 30) was performed to identify similar modules in a hierarchical tree [62]. In total, 45 modules were identified. The module sizes ranged from 30 to 1954. In the network, gene connectivity was based on the edge weight (ranging from 0 to 1) determined by the topology overlap measure. Genes with the highest connectivity inside a module were defined as hub genes. The module eigengene E is defined as the first principal component of a given module. Networks were visualized by Cytoscape software v 3.6.1 [63].

### 4.7. Statistical Analysis

Average and standard derivations of chemicals were calculated using Microsoft Excel software, as were several Figures. Figures were produced and merged by Origin Pro v 8.0 (Origin Lab Corporation, USA). Duncan’s test was applied to detect the significant differences between groups at significant level of 0.05 using SPSS v 19.0 (SPSS Inc., Armonk, NY, USA). Heat map and Partial least squares discriminant analysis (PLS-DA) was carried out by online tool MetaboAnalyst v 4.0 (https://www.metaboanalyst.ca/). Venn diagram was constructed by online tool Venny v 2.1.0 (BioinfoGP Service, Madrid, Spain). The value of variable importance in projection (VIP) was also calculated as a variable to evaluate the contribution of a certain compound. Metabolites whose VIP values exceeding 1.0 were selected for further analysis. The variable identification (VID) coefficients of volatile compounds were calculated using Unscrambler v 10.1 (CAMO Technologies Inc., USA) to identify the most important compounds contributing to sample separation. The VID coefficients were calculated as the correlation coefficient between each original X-variable (volatile compounds) and the Y-variable (*Citrus* species) as predicted by the PLS model. As an arbitrary threshold, a VID coefficient with absolute value of 0.70 was taken [49].

## 5. Conclusions

Our investigation into finger citron metabolite and transcriptome provides new insights into the biosynthesis of terpenoids. In finger citron, expression of upstream genes in the MVA and MEP pathways did not change much during organ development. Terpenoid diversity was caused by differential TPS expression. Based on PLS models, we identified 6 volatiles differentially accumulated in different finger citron organs. Together with the two volatiles with highest contents, these can be considered as the characteristic components of finger citron and can be used to distinguish finger citron from other *Citrus* species. Our results showed there are 58 members of the finger citron *TPS* gene family. The expression patterns of six TF families with 901 members in finger citron organs were also explored. Finally, we obtained 45 gene modules from WGCNA. Based on the high-connectivity subnetwork of Module Red and Module Green, 13 *TPS*s were suggested to play important roles in finger citron characteristic aroma. In addition, 15 TFs were identified whose locations in the subnetwork implied they function in regulating terpenoid metabolism. Future work should focus on the direct and indirect interaction among these target genes and regulators to elucidate the functioning network that controls terpene production.

## Figures and Tables

**Table 1:** The FPKM fold change (FC) of structure genes in terpenoid synthesis during organ development.

Gene ID	Gene Name	FC (F2/F1)	DEG	FC (L2/L1)	DEG	FC (S6/S1)	DEG
**MEP Pathway**							
Cm152440	DXS	1.21		0.49	yes, down	0.20	yes, down
Cm165980	DXS	0.97		1.24		0.12	yes, down
Cm165990	DXS	1.42		2.03		1.34	
Cm219000	DXS	1.09		1.72		1.32	
Cm104830	DXS	0.88		2.08	yes, up	1.49	
Cm018790	DXR	0.95		1.25		0.93	
Cm018780	DXR	1.43		0.86		1.22	
Cm191510	MCT	1.39		0.61		0.90	
Cm162030	CMK	1.34		0.88		0.91	
Cm051360	MDS	0.88		1.60		2.53	yes, up
Cm131750	HDS	1.20		2.67	yes, up	1.52	
Cm315300	HDR	0.31		-		-	
Cm315660	HDR	0.24		-		-	
Cm219660	HDR	0.99		0.80		0.42	yes, down
Cm276630	HDR	1.41		2.10	yes, up	0.93	
Cm318240	HDR	1.05		-		0.61	
**MVA Pathway**							
Cm183050	AACT	0.92		1.03		1.28	
Cm042040	AACT	1.04		0.60		2.90	yes, up
Cm014300	HMGS	1.17		0.82		1.24	
Cm248930	HMGS	0.98		0.38	yes, down	1.47	
Cm197870	HMGR	0.93		0.91		1.42	
Cm212470	HMGR	0.72		0.86		0.66	
Cm122170	HMGR	1.56		0.33	yes, down	5.51	yes, up
Cm268880	MVK	1.23		0.97		0.55	
Cm034040	PMK	1.10		0.52		1.08	
Cm235330	MVD	1.13		0.55		0.65	

Genes whose log2(FC) ≥ 1 and *p* < 0.05 were considered as differentially expressed genes (DEGs). AACT, Acetoacetyl-CoA thiolase; HMGS, HMG-CoA synthase; HMGR, HMG-CoA reductase; MVK, mevalonate kinase; PMK, phosphomevalonate kinase; MVD, mevalonate diphosphate decarboxylase; FPPS, farnesyl diphosphate synthase; DXS, 1-deoxy-D-xylulose 5-phosphate synthase; DXR, 1-deoxy-D-xylulose 5-phosphate reductoisomerase; MCT, 4-diphosphocytidyl- 2-C-methyl-D-erythritol synthase; CMK, 4-diphosphocytidyl-2-C-methyl-D-erythritol kinase; MDS, 2-C-methyl-D-erythritol 2,4-cyclodiphosphate synthase; HDS, 4-hydroxy-3-methylbut-2-enyl diphosphate synthase; HDR, 4-hydroxy-3-methylbut-2-enyl diphosphate reductase.

## Supplementary Materials

The following are available online. Table S1: Content (μg/g FW) of 62 identified volatiles in finger citron; Table S2: Summary of the Finger Citron Transcriptome; Table S3: Summary of TF family gene ID. Table S4: Gene ID and annotations in Module Red. Table S5: Gene ID and annotations in Module Green. Table S6: Primer pairs used in qRT-PCR validation. Table S7: The expression profile of assembled genes in RNA-Seq. Figure S1: Results of permutation tests for PLS-DA using volatiles as variables; Figure S2: Pearson correlations between tested samples based on RNA-Seq data; Figure S3: Expression levels of genes based on RNA-Seq data; Figure S4: Schematic diagram of finger citron *TPS* gene structure. Figure S5: Expression analysis of finger citron TPS genes using RNA-Seq and RT-qPCR. Figure S6: Hierarchical clustering and heat map visualization of changes in transcription factor expression level in developing organs of finger citron; Figure S7: Module Red and Module green coexpression networks; Figure S8: Gene co-expression subnetwork of Module Blue and gene annotations; Figure S9: Amino acid sequence similarity of finger citron TPSs and functional characterized TPSs from other *Citrus* species.
