# Supplementary material for: Integration of Metabolite Profiling and Transcriptome Analysis Reveals Genes Related to Volatile Terpenoid Metabolism in Finger Citron (C. medica var. sarcodactylis)
Source: Molecules. 2019 Jul 15;24(14):2564. doi: 10.3390/molecules24142564 (PMC6680504; doi:10.3390/molecules24142564)
Supplement: Supplementary file 1 [file molecules-24-02564-s001.zip › Revised Supplementary files/Supplemetary figure.docx]

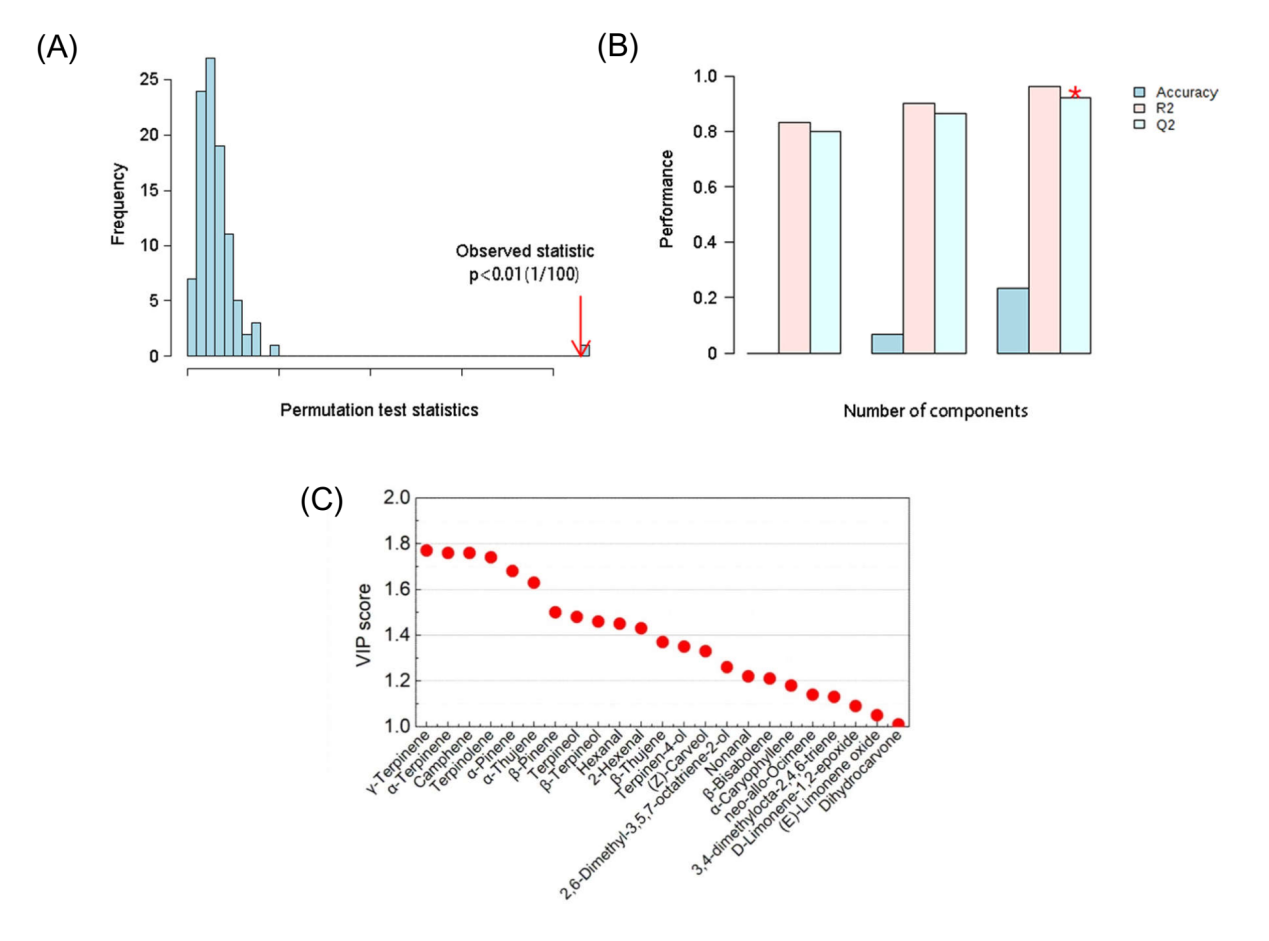


**Figure S1.** Results of permutation tests for PLS-DA using volatiles as variables.

(A) Results of permutation tests for PLS-DA using volatiles as variables. (B) Cross validation test. (C) Volatiles ranked by VIP scores. PLS-DA was carried out by MetaboAnalyst 4.0.


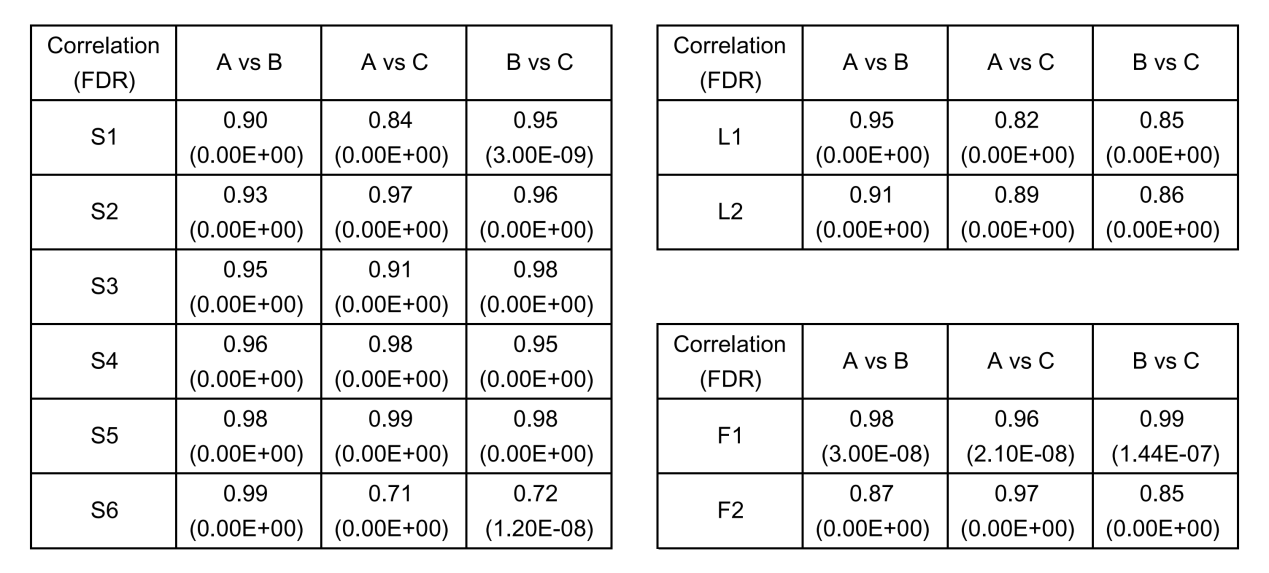


**Figure S2.** Pearson correlations between tested volatile samples based on RNA-seq data.

The correlation between biological replicates for each sample was calculated. The corresponding false discovery rates（FDR）was presented in brackets.


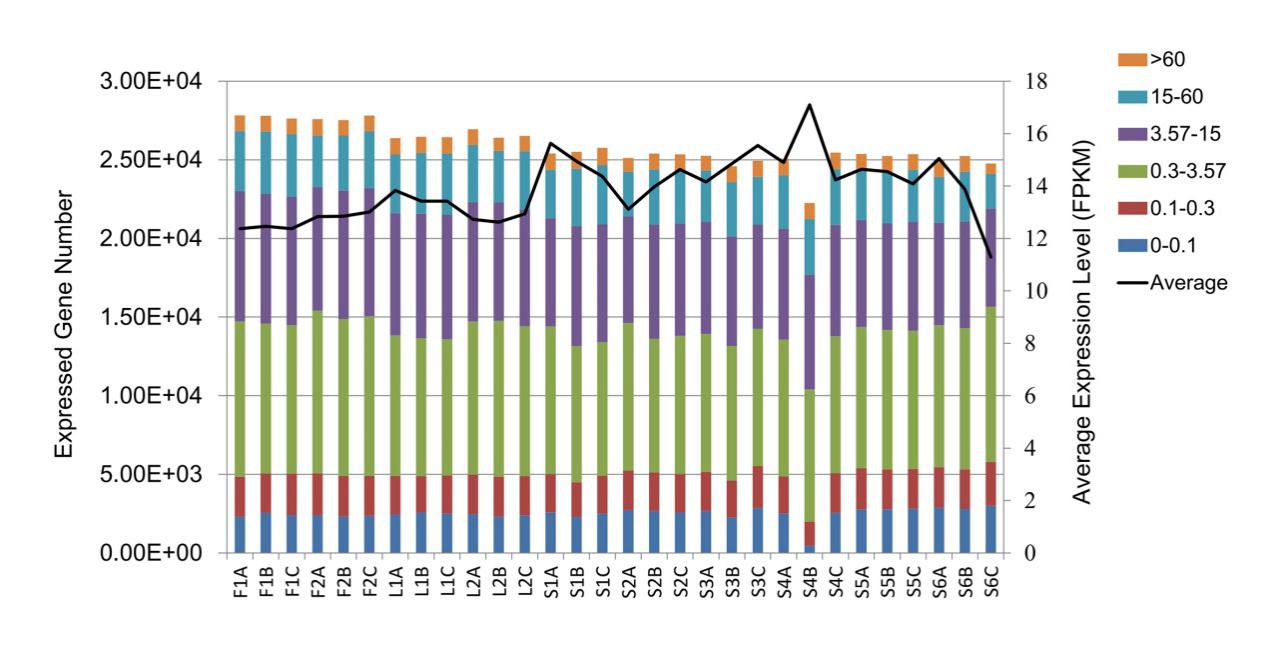


**Figure S3.** Expression levels of genes based on RNA-Seq data.

Left axis represents number of genes, right axis represents FPKM value.


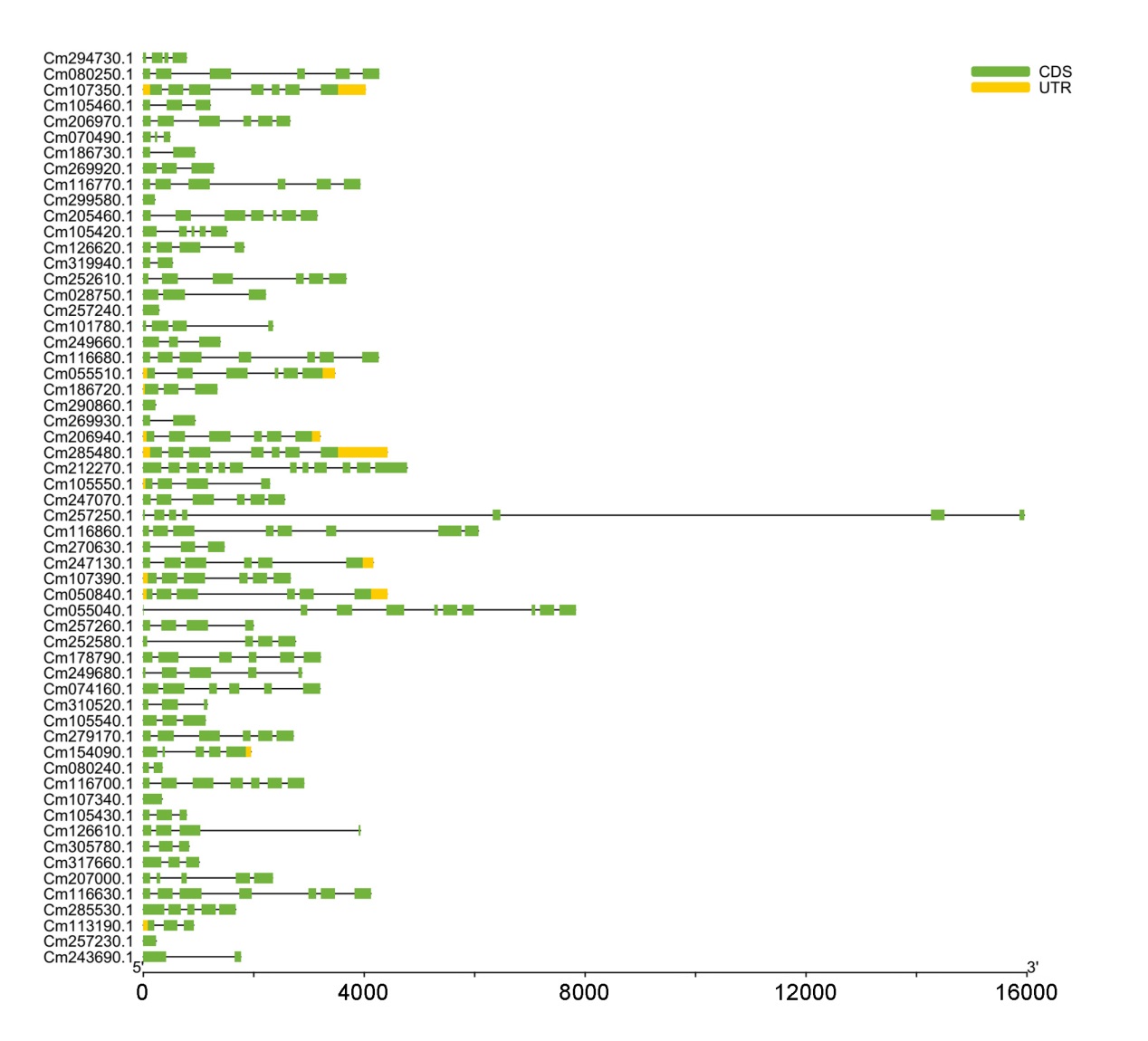


**Figure S4.** Schematic diagram of finger citron TPS gene structure.

Green boxes represent exons, yellow boxes represent untranslated region, black lines represent intron.


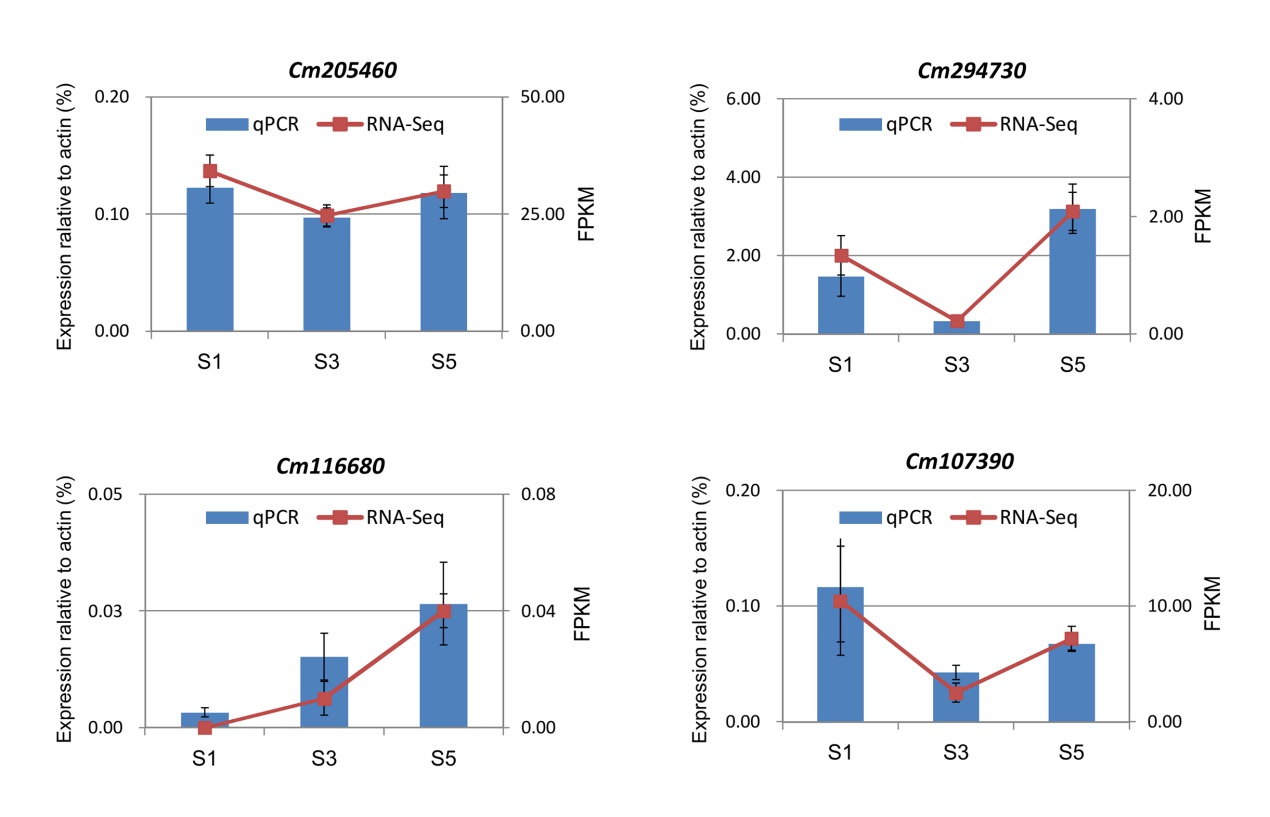


**Figure S5.** Expression analysis of finger citron TPS genes using RNA-Seq and RT-qPCR.

Quantification of expression levels by qPCR analysis normalized to equal levels of actin transcripts. Each value represents the mean ± SE of three replicates.


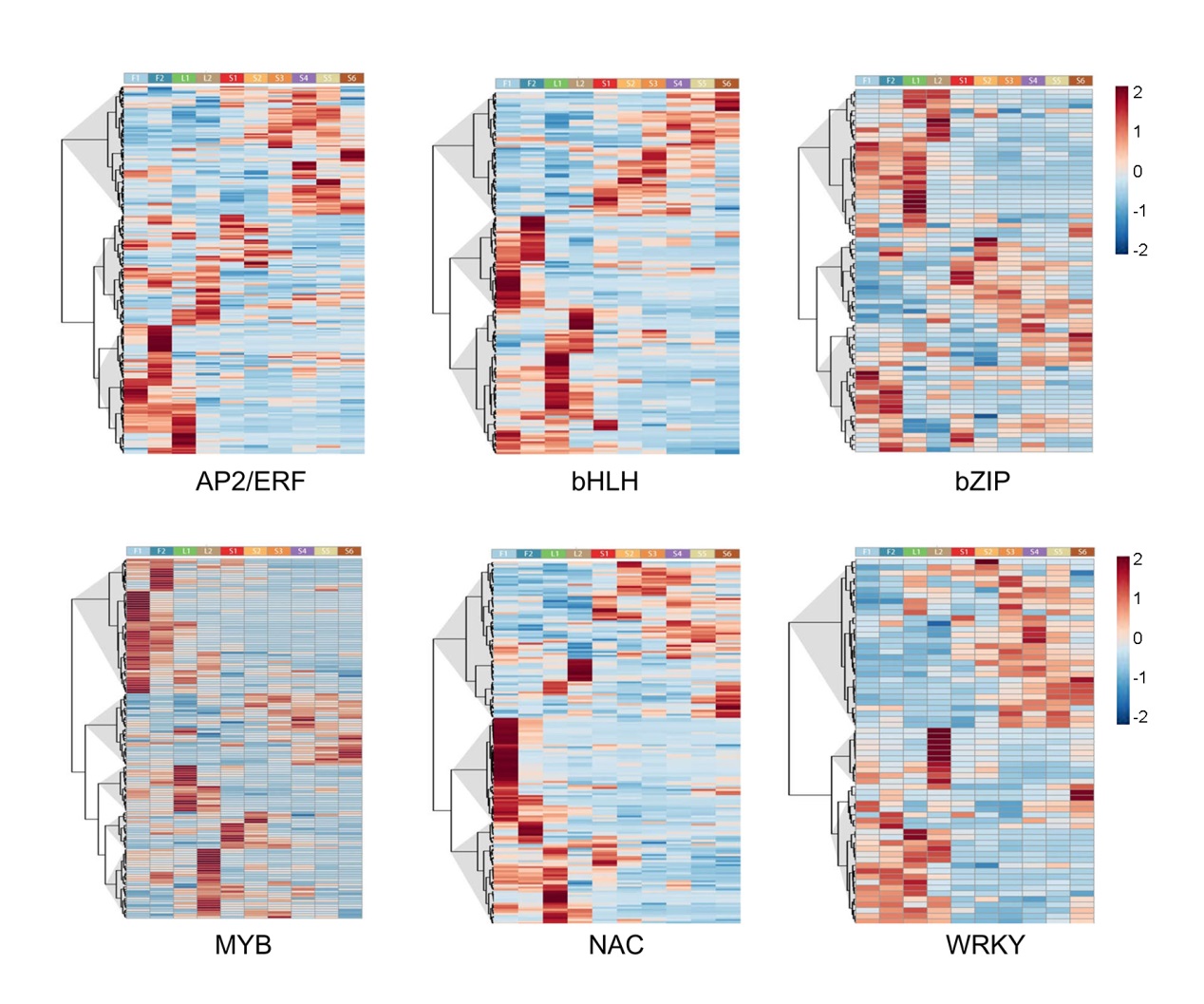


**Figure S6.** Hierarchical clustering and heat map visualization of changes in transcription factor expression level in developing organs of finger citron.

F1, flower bud; F2, full flower, L1, young leaf; L2,mature leaf; S1-6, fruit developing stages 1-6. The left side of the heat map represents hierarchical clustering based on Pearson correlation. The color scale (-2 to 2) is shown on the right; red represents high content, blue represents low content. Hierarchical clustering and heat map were constructed using MetaboAnalyst 4.0.


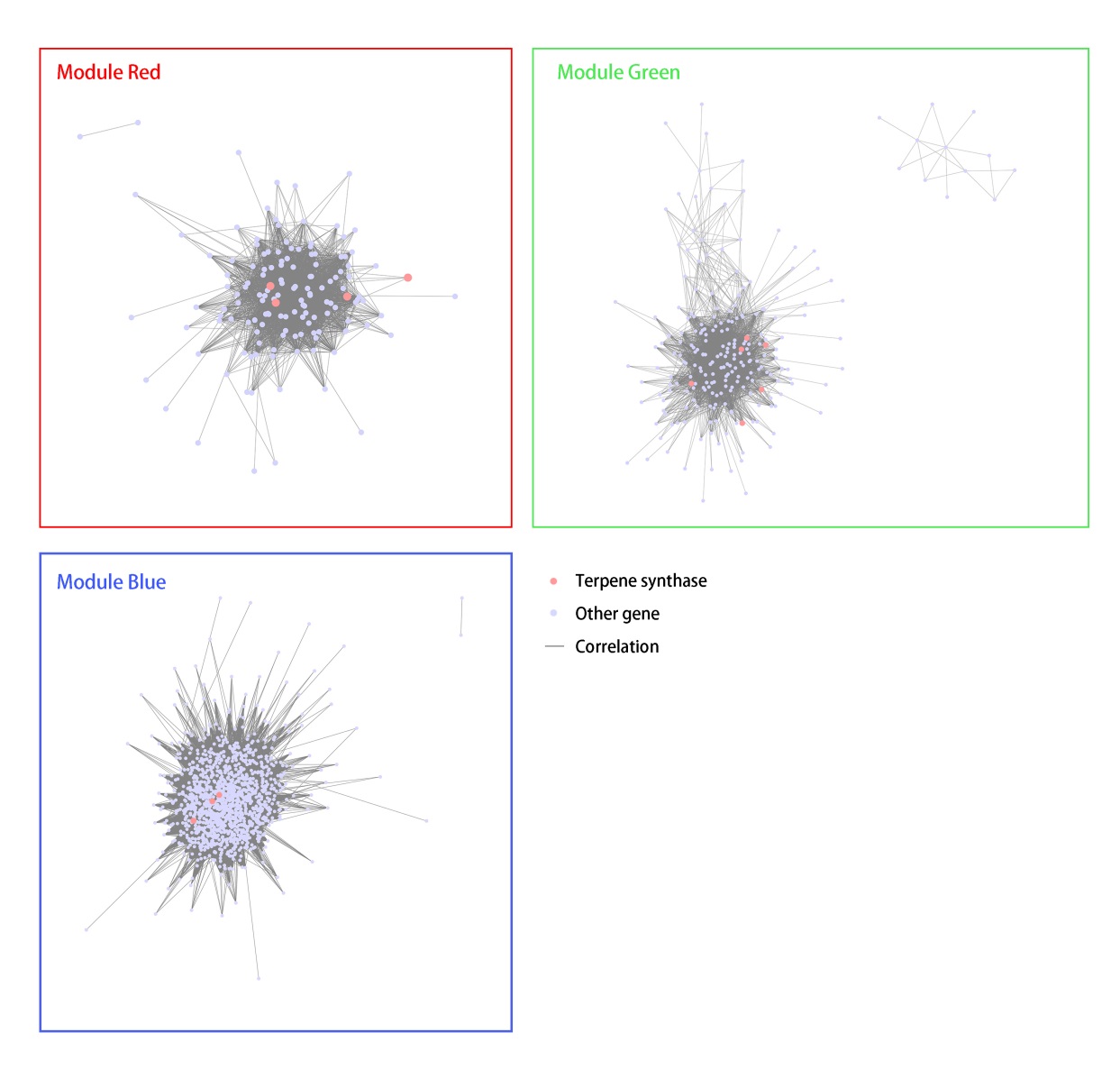


**Figure S7.** Module Red and Module green coexpression networks.

Red nodes represent TPS genes, blue nodes represent TFs. Networks are reconstructed by edge weight cutoff =0.35.


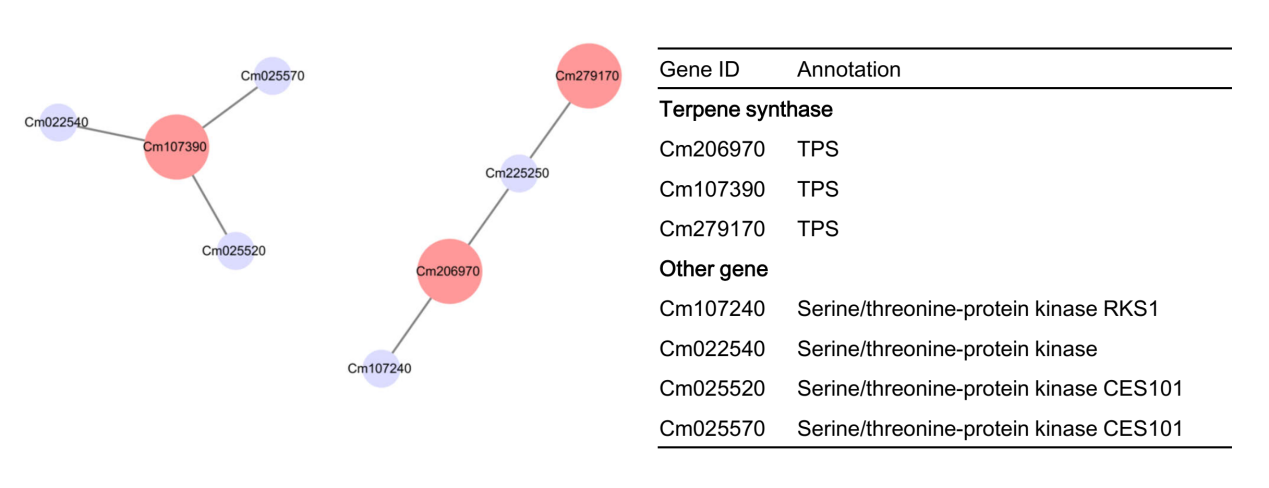
**Figure S8.** Gene co-expression subnetwork of Module Blue and gene annotations.

Network was reconstructed by edge weight cutoff = 0.35 and visualize by cytoscape. Gene IDs and annotations were listed on the right.


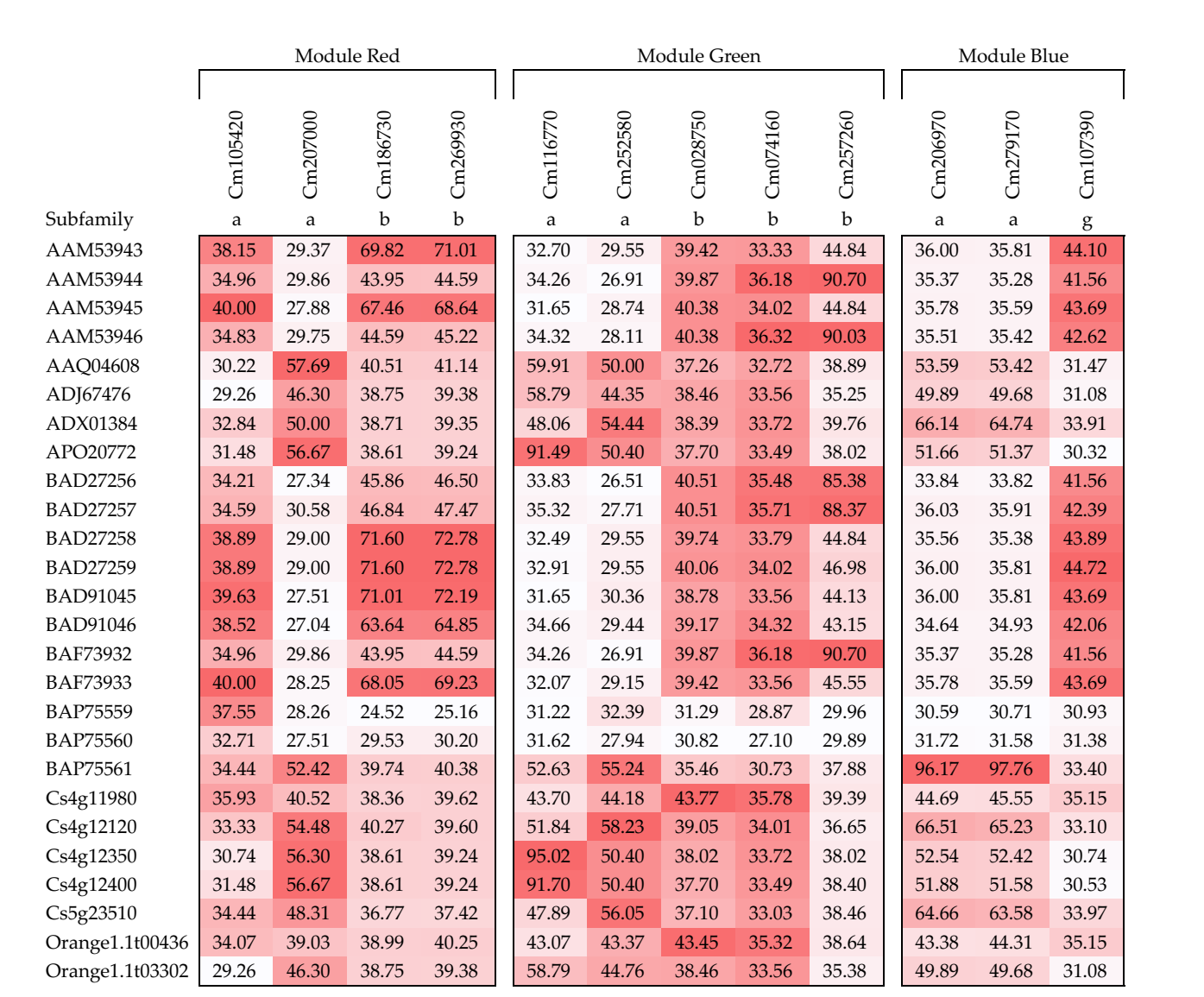


**Figure S9.** Amino acid sequence similarity of finger citron TPSs and functional characterized TPSs from other *Citrus* species.

AAM53943, *C. limon* γ-terpinene synthase; AAM53944, *C. limon* (+)-limonene synthase; AAM53945, *C. limon* (-)-β-pinene synthase; AAM53946, *C. limon* (+)-limonene synthase; AAQ04608, *C. sinensis* valencene synthase; ADJ67476, *C. reticulata* (*E*)-β-farnesene synthase; ADX01384, *C. hystrix* Germacrene D synthase; APO20772, *C. medica* var. *sarcodactylis* bicyclogermacrene synthase; BAD27256, *C. unshiu* d-limonene synthase; BAD27257, *C. unshiu* d-limonene synthase; BAD27258, *C. unshiu* γ-terpinene synthase; BAD27259, *C. unshiu* γ-terpinene synthase; BAD91045, *C. unshiu* monoterpene synthase; BAD91046, *C. unshiu* (E)-beta-ocimene; BAF73932, *C. jambhiri* limonene synthase; BAF73933, *C. jambhiri* β-pinene synthase ; BAP75559, *C. unshiu* linalool synthase; BAP75560, *C. unshiu* linalool synthase; BAP75561, *C. unshiu* linalool synthase. *C. sinensis* sesquiterpene synthases: Cs4g11980, Cs4g12120, Cs4g12350, Cs4g12400, Cs5g23510, Orange1.1t00436 and Orange1.1t03302.
